# Compensatory and Lifestyle-Based Brain Health Program for Subjective Cognitive Decline: Self-Implementation versus Coaching

**DOI:** 10.3390/brainsci11101306

**Published:** 2021-09-30

**Authors:** Harris Liou, Cynthia M. Stonnington, Amit A. Shah, Skye A. Buckner-Petty, Dona E. C. Locke

**Affiliations:** 1 Alix School of Medicine, Mayo Clinic, Scottsdale, AZ 85259, USA; liou.harris@mayo.edu; 2 Department of Psychiatry & Psychology, Mayo Clinic, Scottsdale, AZ 85259, USA; stonnington.cynthia@mayo.edu; 3 Department of Community Internal Medicine, Mayo Clinic, Scottsdale, AZ 85259, USA; shah.amit@mayo.edu; 4 Research Services, Mayo Clinic, Scottsdale, AZ 85259, USA; buckner-petty.skye@mayo.edu

**Keywords:** subjective cognitive decline, brain health, compensatory training

## Abstract

Although recent studies have explored the potential of multidomain brain health programs, there is a dearth of literature on operationalizing this research to create a clinical treatment program specifically for subjective cognitive decline (SCD). Patients seen by geriatricians in primary care and by behavioral neurology services at our institution presenting with SCD were recruited via a patient-appropriate flyer. After all participants had a 1-h brain health consultation with a neuropsychologist and were provided with program materials, they were randomized to attend a 10-week intervention designed to support program implementation (N = 10) or the control group of implementing the program on their own (N = 11). The program included (1) a calendar-based executive and memory support system for compensatory training and (2) training in healthy lifestyle. There were no significant differences between groups for any outcomes. Participants across both groups showed significant improvements with moderate effect sizes in compensatory strategy use, anxiety symptoms, and daily functioning, which were sustained through 6-month follow-up. They also increased physical activity by the end of the intervention period but did not sustain this through 6-month follow-up. Our pilot study demonstrates preliminary feasibility of a cognitive compensatory and lifestyle-based brain health program. Additional research is recommended to further develop two potentially scalable implementation strategies—coaching and self-implementation after brief consultation.

## 1. Introduction

As the population of older adults rises, there is increasing need to address the public health crisis of age-associated cognitive impairment. Although pharmaceutical treatment options are severely limited, there is a growing body of evidence for behavioral interventions that can prevent or delay the onset of dementia [1]. As such, there has been growing provider and public interest in the concept of brain health, which is the lifelong maintenance of optimal cognitive, emotional, psychological, and behavioral function [2]. According to a recent survey, 90% of individuals believe their doctors should monitor cognitive health but feel they do not receive the brain health care they expect [3]. Thus, there is a gap in healthcare concerning preventative brain health measures.

Traditionally, mild cognitive impairment (MCI) has been the objectively-testable diagnosis used to describe patients in a transitional state between normal aging and dementia, most often due to Alzheimer’s disease (AD) [4]. To standardize the emerging research on the earliest stages of AD, the working group of the Subjective Cognitive Decline Initiative (SCD-I) recently defined subjective cognitive decline (SCD) as subjective and/or subtle objective cognitive decline not meeting criteria for MCI [5]. This typically presents in the primary care setting as self-reported confusion or memory loss but without significant impairments in daily functioning and age-appropriate performance on mental status testing. Posing a significant public health issue, SCD currently affects 11.1% of adults in the U.S. and will likely increase in prevalence [6].

A longitudinal study by Farias et al. demonstrated relatively linear progressions in everyday functional impairment among subjects who had normal cognitive function at baseline and developed MCI or dementia [7]. Indeed, more than half of adults with SCD have experienced mild functional changes in tasks such as paying bills, taking medications, and driving [6]. Such decline is of great concern at every stage of normal aging or early stages of pathology, as preservation of autonomy and independence are among the top priorities of older adults [8]. Furthermore, a meta-analysis found that 27% and 14% of individuals with SCD progressed to MCI and dementia, respectively [9]. Studies have also shown that SCD is associated with biomarkers of early AD such as increased Amyloid-β [10] and neurodegeneration [11]. Thus, SCD patients are an at-risk group with a critical window of opportunity to both ameliorate the effects of SCD and prevent or delay the onset of progressively debilitating functional impairment.

Subjective changes in memory and executive function are some of the earliest signs of evolving MCI that influence daily functioning and are thus important targets for intervention [12,13]. Compensatory strategies empower patients to adapt to the various impairments posed by their illness. A promising application of such interventions to help patients cope with memory loss is the Memory Support System (MSS) developed by Greenaway et al. The system consists of a pocket-size calendar and note taking system on which patients log appointments and “to do” lists as well as maintain a journal [14]. When administered with appropriate training, the MSS improved functional ability, memory self-efficacy, and mood in patients with MCI [15]. Another compensatory strategy is Goal Management Training^®^ (GMT), which is a metacognitive rehabilitation program designed to improve executive function by teaching patients to periodically stop what they are doing, think about task goals, and evaluate their performance [16]. A GMT trial on SCD patients demonstrated improvements in performance on simulated real-life tasks and self-rated executive deficits coinciding with training [17].

Ideally, patients would be able to employ these compensatory strategies to not only function more efficiently in daily activities, but also implement sustained lifestyle modifications to further enhance brain health. Many studies have demonstrated the positive impact of physical exercise on cognition and brain health [18,19]. A recent meta-analysis of exercise interventions for cognitive function in older adults showed that physical exercises including aerobic exercise, resistance training, multicomponent activity, and tai chi improved cognitive function [20]. The benefit was seen both in subjects with MCI and subjects without objective cognitive impairment. Similarly, there is increasing evidence for the association of engagement in cognitively stimulating activities with better cognitive function in old age [21,22]. Among the many translational studies on this subject, Smith et al. demonstrated that adaptive computerized cognitive training improves attention and memory as well as self-reported daily functioning [23]. Lastly, stress and poor mental health can also impact cognition and brain health [24]. For example, chronically elevated stress can lead to structural brain changes due to prolonged activation of complex hormonal systems [25]. A potential intervention to address this brain health hazard is meditation and mindfulness, which has been shown to offset age-related cognitive decline [26].

Recent studies have investigated the potential to synthesize the numerous discreet reports on individual interventions that demonstrate brain health protection into multidomain clinical programs based on behavioral change. For example, the Finnish Geriatric Intervention Study to Prevent Cognitive Impairment and Disability (FINGER) tested a program including diet, exercise, cognitive training, and vascular risk monitoring in older adults with cognitive performance at or slightly below levels expected for age and reported significantly higher neuropsychological test battery scores in the intervention group compared to the control group receiving general health advice [27]. Moreover, Denny et al. designed a coaching intervention incorporating compensatory training encompassing use of a daily planner system and GMT as well as lifestyle changes that demonstrated significantly increased compensatory behaviors, engagement in cognitive activities, and meditation in patients with SCD compared to waitlist controls [28]. Despite the need for early intervention before MCI and all the potential interventions with promising preliminary data, there is a dearth of literature on operationalizing this emerging research to create a clinical treatment program specifically for SCD. Currently, there is no consensus regarding the standard of care for SCD, and providers are to simply monitor for comorbidities that may impair memory as well as any decline of objective cognitive performance [29].

The goal of this study is to begin a path towards bridging this gap in care and pilot an evidence-based and scalable treatment option for SCD. We expanded upon the intervention piloted by Denny et al. [28] to test a person-centered program based on well-established theories of behavioral change and self-efficacy [30,31]. The program, titled the Executive and Memory Support (EMS) system, is designed to (1) bolster the consistent and effective use of compensation strategies that support everyday memory and executive functioning utilizing a specific daily planner system (the MSS) and (2) utilize these strategies to promote adopting, enhancing, and maintaining engagement in activities that have been shown to promote brain and overall health and daily well-being. In this trial, Our primary objective was to assess feasibility and scalability of this program by whether and how patients would implement these tools in their daily lives by comparing self-implementation to group coaching. We predicted that those who were coached would be more likely to incorporate these behaviors into their daily routine than those who just received the consultation. We intentionally utilized a real-world setting in line with the movement toward pragmatic clinical trials (see https://www.nia.nih.gov/research/blog/2017/06/pragmatic-clinical-trials-testing-treatments-real-world, accessed 27 September 2021).

## 2. Materials and Methods

### 2.1. Participants

Patients seen by the geriatricians in primary care and the behavioral neurology service at Mayo Clinic Arizona who expressed cognitive concerns but had no evidence of objective cognitive decline were presented with a patient-appropriate recruitment flyer describing the trial by their providers. They were asked permission to send their contact information to the trial coordinators for further discussion of the research program. Those who agreed to be contacted by the research coordinator were then screened based on the following criteria, after which 21 participants were enrolled. This study was approved by the Mayo Clinic IRB and all participants signed a written informed consent for prior to initiation of any eligibility screening, data collection, randomization, or intervention.

Inclusion Criteria:

(1) Age 50 or older
(2) A positive complaint or concern in response to two questions: “Do you feel like your memory or other aspects of thinking are becoming worse?” and “Does this worry you?”
(3) Normal cognitive performance on the Montreal Cognitive Assessment [32] (adjusted for age and education)
(4) Self-reported independent function in daily life as measured by the Lawton IADL scale (IADL = 8) [33]
(5) English speaking
(6) Approval letter from a physician (due to exercise component)

Our inclusion criteria conformed to the Subjective Cognitive Decline Initiative Working Group standards and is consistent with other large-scale studies [5,34,35].

Exclusion Criteria:

(1) Known neurological disorder with potential cognitive symptoms (Parkinson’s disease, epilepsy, history of traumatic brain injury, etc.)
(2) Uncontrolled moderate or severe depression (e.g., CES-D > 21)

### 2.2. Intervention

All participants were provided with a 1-h brain health consultation with a neuropsychologist (DCL) as well as program materials. Materials included a copy of the Memory Support System planner, a subscription to the BrainHQ cognitive exercise platform (by Posit Science), and a recommendations document (see Supplemental Materials). This document included, for each component of the program, a 1-page description of the component, recommendations for how to approach the associated tasks, and a specific goal for the participant to aim for. Components included all those covered in the coaching classes (the MSS, task lists, functional zones, physical exercise, cognitive exercise, and stress management/mindfulness).

After the 1-h consultation, participants were then randomized to attend a 10-week coaching-class intervention designed to support program implementation or to the active control group of implementing the program on their own. We used block randomization with 1:1 matches using the nearest neighbor matching on age, gender, and adjusted MOCA score. We had some couples who wanted to participate in the study together so we added the constraint that spouses cannot be matched to each other. Each matched pair was selected in random order. If a matched pair contained a participant who was the spouse of an already randomized subject, then that participant was assigned to the same treatment arm as his/her spouse and the other member of the matched pair was assigned to the opposite treatment arm. Otherwise, the members of the matched pair were randomly assigned to either arm with equal probability.

The coaching-class was administered over 10 weeks in 2-h weekly sessions provided in a group class format. Weekly sessions featured a new technique or behavior, but the last three weeks were for review focused on skill building and practice. Each class followed a similar format: a brief review of class material and homework, new class material (including group discussion and in-class activity), and discussion of homework for the coming week. The program included 2 main components: (1) the Executive and Memory Support (EMS) System for compensatory training and (2) training in healthy lifestyle. More details of each component and how they were covered in the coaching classes is described next.

#### 2.2.1. The Executive and Memory Support (EMS) System

The EMS system included three training components:

(1) Planner System (The “Memory Support System” (MSS) [14,15]):

The calendar system included a 2-page per day calendar/note-taking system for the current month and a monthly-view annual calendar for the year-at-a-glance. It was small enough to fit in a breast pocket or purse. The 2-page per day section had three subsections: (1) a daily calendar where events/appointments were recorded on one page and (2) a daily “to do” list as well as (3) a journaling section on the second page. The annual calendar section included a month-at-a-glance view with additional journaling/notes pages. This journaling section was where short- and long-term goals were maintained and updated (see Goal Planning below), which were also to be incorporated into the daily “to do” task list. Training followed well-established learning theory and rehabilitation methods that incorporated an initial training/acquisition phase (in-class training session), followed by an application and adaption phase utilizing practice and feedback [36]. A key component was for participants to make a habit of (1) reviewing the calendar routinely (i.e., designated times daily) and (2) entering all re-occurring and non-reoccurring activities. Class discussions focused on barriers and resistance. Homework included entering specified activities into the MSS.

(2) Goal Planning

While the original MSS calendar system included a section for a “to do” list, it did not incorporate a formalized curriculum for aspects of executive functioning, such as goal planning and management. This second compensatory component employed GMT to help participants identify short- and long-term goals, plan and prioritize steps to accomplish goals, break goals into manageable and sequential steps, write them down in the MSS annual calendar journaling section, and transfer steps to the daily “to do” section of the MSS. Individualized goal-setting served to motivate participants and provided a “behavioral contract”. Class discussion/homework subdivided goals and used the daily “to do” list.

(3) Organization Systems for the Environment

This final compensatory component focused on structuring and organizing the environment effectively and efficiently to accomplish daily tasks. Participants parsed their physical environment and life space into “functional zones” associated with specific daily activities (e.g., home office, kitchen, exercise space). Discussion topics included the benefits of well-organized zones (e.g., finding things easily saves time), identifying functional zones, and developing plans for better organization of selected zones. Homework focused on reorganizing personal functional zones.

#### 2.2.2. Training in Healthy Lifestyle

A unique advantage of this intervention was the use of compensation strategies in adopting brain-healthy lifestyles. Participants set goals related to these activities, utilized the MSS “to do” list or scheduled activities at specific times, and used organizational strategies to support and sustain these habits.

(1) Physical Exercise

Psychoeducation was provided about the impact of regular aerobic exercise on cognitive/brain health and participants were taught how to monitor heart rate. Rather than adhering to a standardized exercise regime, participants developed an individualized physical activity plan. A list of specific options of physical activities was provided and discussed. Group classes involved discussion of barriers to exercise and solutions for overcoming those barriers. For homework, participants engaged in physical activities at least 150 min per week. Physical activities were scheduled into participants’ calendars or included in the “to do” lists in the MSS.

(2) Cognitive Stimulation

Psychoeducation was provided on the benefits of cognitive stimulation. Emphasis was placed on engaging in activities that required novelty, challenge, speed, and sustained processing. Participants were given a one-year subscription to BrainHQ, an adaptive computerized cognitive training program utilized in prior research in cognitive aging [37,38]. In addition, participants were presented with a spectrum of cognitive activities (e.g., juggling, learning a musical instrument, dancing) and identified an individualized set of activities to engage in each week. Group classes involved discussion of barriers to exercise and solutions for overcoming those barriers. For homework, participants engaged in stimulating activities for at least 150 min per week. Cognitive activities were also scheduled into participants’ calendars or included in their “to do” lists in their MSS.

(3) Stress management

Information was provided on the contribution of depression, anxiety, and other forms of emotional distress to reduced brain health. Participants were also educated on the physiological stress response. Stress management utilized sitting meditation, mindful movement, and the body scan, which had been previously demonstrated to be implementable [39,40]. For homework, participants engaged in meditative practices or mindfulness exercises at least 4×/week for at least 15 min at a time. Meditation or mindfulness exercises were also scheduled into participants’ calendars or included in their “to do” lists in their MSS.

### 2.3. Outcomes

All outcomes (described below) were measured at baseline (before participants were provided program materials), immediately post-intervention (or 10 weeks after participants were provided program materials for the self-implementation group), and at 3- and 6-month follow-up points. Of note, the intervention was completed in March of 2020, so the 3- and 6-month follow-up points occurred during the COVID-19 pandemic public health emergency declaration.

#### 2.3.1. Primary Outcomes

Compensation metric: The Everyday Compensation Questionnaire [41] (EComp) was collected both from participants and informants when available. It measures various types of compensation strategies used when completing specific activities of daily living that were taught as part of the EMS System (e.g., carrying a calendar at all times, using a calendar to track appointments, keeping the home office organized, preparing ahead for an event, using an organized system for medications). The EComp has good reliability (e.g., Cronbach’s alpha = 0.91) and is related to objective measures of cognition. In addition, we measured MSS usage as defined by Greenaway et al. [14]. Usage scores range from 0–10 based on the following criteria: 1) bringing the MSS to the appointment (1 point); having at least 1 entry for today’s date (1 point); picking 2 days at random and having 1 entry in each “appointments” section (maximum 2 points), 1 entry in each “to do” section (maximum 2 points); and 2 entries in each journaling/notes section (maximum 4 points).

Cognitive activity ratings: The Lifestyle Activities Questionnaire [42] (LAQ) was completed by participants. The LAQ is a 23-item questionnaire measuring frequency of participation in cognitively stimulating activity (e.g., reading, playing games of skill, visiting museums). In addition, time engaged with BrainHQ was tracked via the BrainHQ portal.

Physical activity ratings: A 41-item physical activity (PA) questionnaire was completed by participants to capture self-reported PA [43,44]. Participants listed frequency and time spent weekly in each activity.

Mindfulness: The Mindful Attention Awareness Scale [45] (MAAS) was completed by participants. The MAAS is a 15-item measure of mindfulness where higher scores indicate more mindfulness.

#### 2.3.2. Secondary Outcomes

Everyday function: When an informant was available, the Everyday Cognition [46] (ECog) was used to measure functional abilities across six cognitively relevant domains: everyday memory, language, visuospatial abilities, planning, organization, and divided attention. The ECog has been extensively validated and correlates highly with objective cognitive test scores, performance-based measures of function, and biomarkers of disease. Test-retest reliability over an average of 29 days is good (r = 0.82).

Quality of Life: Quality of life was measured using the Quality of Life-Alzheimer’s Disease (QOL-AD) scale [47].

Sense of Purpose: Sense of Purpose was measured using Ryff’s scale of psychological well-being [48].

Depression: Participants completed the Centers for Epidemiological Studies-Depression measure [49] (CES-D).

Anxiety: Participants completed the Anxiety Inventory Form (AIF), a 10-item rating scale modified from the State-Trait Anxiety Inventory by the Resources for Enhancing Alzheimer’s Caregiver Health (REACH) project [50].

Cognition: Memory was measured using the Hopkins Verbal Learning Test [51] (HVLT), executive function using the Controlled Oral Word Association Test [52] (COWAT), and psychomotor speed using Symbol Digits [53]. These tasks were administered to the subject by a psychometric technician.

### 2.4. Statistical Analyses

An a priori power analysis was not performed, as this trial was designed as a pilot study. We first aimed to track enrollment as well as completion of the intervention, especially in the coaching class condition. Our planned primary outcome measure analysis was comparison of change in the EComp, LAQ, PA, and MAAS between groups at each time point utilizing paired *t*-tests. All other analyses were considered secondary and exploratory. In the comparisons where treatment groups were combined, primary, secondary, and cognitive outcome measures assessed at three follow-up timepoints were compared to the baseline measures via paired *t*-test. Cohen’s D effect sizes were also computed for the magnitude of change in each measure. Differences in the change scores of the primary outcomes were assessed with Student’s *t*-tests. *p*-values less than 0.05 were considered statistically significant. All analyses were completed using R version 3.6 (R Core Team, 2019).

## 3. Results

**Figure 1:**
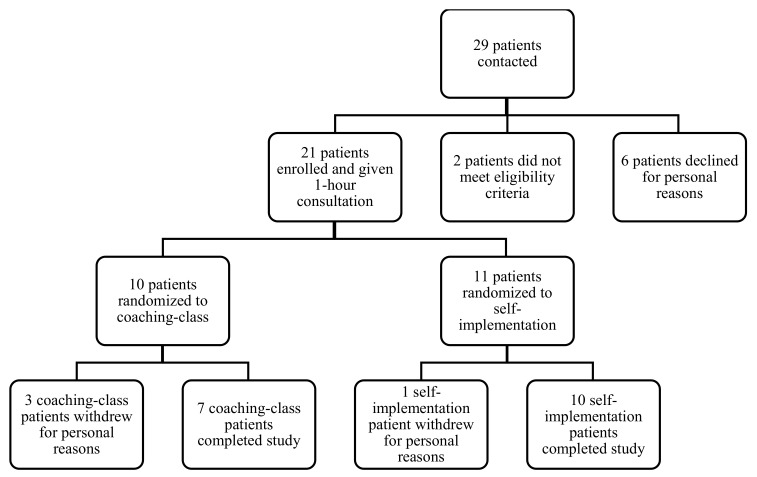
Patient Participation and Randomization.

Participation of patients is outlined in Figure 1. Of the 29 patients who provided their contact information to learn more about the trial and were subsequently reached, 21 were enrolled in the study. There were 2 patients who did not qualify based on eligibility criteria (1 due to cognitive impairment and 1 due active severe depression). After contact, 6 patients declined participation in the study due to time constraint in the event of randomization to the coaching-class group (4 patients) and traveling during the study period (1 patient); 1 patient did not provide a reason. Of the enrolled patients, 10 were randomized to the coaching-class group and 11 to the self-implementation group. One self-implementation participant withdrew from the study due to a family member being placed in hospice, and 3 coaching-class participants withdrew because of surgery, scheduling conflicts, and lost interest. In the coaching-class group, participants attended 85.7% of all classes and completed a median of 80% of assigned homework. Table 1 lists demographics of the remaining active participants. There were no significant differences in any demographics between the coaching-class and self-implementation groups.

### 3.1. Primary Outcome Measures

There were no statistically significant differences between the coaching-class and self-implementation groups for change of the EComp, PA, LAQ, or MAAS measures at any time point (*p* = 0.32 to 0.77 across measures and time points; See Supplemental Material). For MSS usage, there was a trend toward greater use in the coaching group (mean = 6.86, SD = 2.5) compared to the self-implementation group (mean = 3.7, SD = 4.0) at the end of the treatment period (*p* = 0.06, d = 1.03). This difference was not sustained at the 3-month or 6-month follow-ups. There were 3 in the self-implementation group and 1 in the coaching-class group who opted not to start using the MSS at all. With those four participants removed from the usage measurement, the difference between groups was significant at treatment end (coaching mean = 8.0, SD = 2.9; self-implementation mean = 4.9, SD = 2.0; *p* = 0.04, d = 1.32). For BrainHQ usage, there were no significant differences between groups, but raw score differences favored the self-implementation group. By the end of the intervention period (10 weeks after trial initiation), participants in the self-implementation and coaching class groups spent on average 190 (median 150, range 0–524) and 58 (median 57, range 0–182) hours, respectively, on BrainHQ (*p* = 0.22). Self-implementation and coaching participants spent on average a total of 517 (median 399, range 0–1796) and 97 (median 60, range 0–428) hours, respectively, by the 6-month follow-up (*p* = 0.16). There were 2 self-implementors and 2 coaching participants who never activated their Brain HQ accounts.

Given no differences between groups on primary measures and the pilot and exploratory nature of this study, we also conducted secondary analyses with the treatment groups combined. We aimed to explore if there was any suggestion of change with intervention as a whole that could be further pursued in follow-up studies, in the spirit of pragmatic clinical trial development. There was significant change from baseline to study end across the groups combined, which is detailed in Table 2. Both groups showed an increase in PA with a moderate effect size at intervention period end. Unfortunately, this increase was not sustained through the 6-month follow-up. There was also improvement in self- and informant-reported use of compensatory strategies in the sample as a whole, which was sustained through the 6-month follow-up. There were no significant changes for the sample as a whole on MAAS or the LAQ.

### 3.2. Secondary Outcomes

Similar to the primary outcomes there are significant improvements from baseline with moderate effect sizes for the groups combined (Table 3). Specifically, both groups showed sustained reduction in anxiety symptoms at 6 months and informants reported sustained improvement in daily functioning at 6 months.

### 3.3. Cognitive Outcomes

This project was started in January of 2020. Therefore, the three-month and six-month follow-up visits were unexpectedly impacted by the COVID pandemic. We were able to collect the self and informant report measures via electronic survey, but because cognition measurements (HVLT, COWAT, and Symbol Digits) must be administered in person, many participants elected not to undergo cognition tests after baseline. The limited results are listed with effect sizes in Table 4, in which there were significant improvements in HVLT total recall, COWAT, and Symbol Digits among all subjects. This likely represents exposure practice effects.

## 4. Discussion

Currently the standard of care for patients with SCD is to reassure and follow. We conceptualized our pilot study as a step toward testing the feasibility of an expanded program that empowers patients to take action and instill behaviors that promise to preserve function by compensating for subjective or objective cognitive decline. By evaluating two mechanisms for implementation of compensatory and brain health behaviors after consultation with a neuropsychologist (self-implementation and coaching classes), we add to the original study by Denny et al. [28] that demonstrated the efficacy of the program via a one-arm coaching study. Further differences in study design included the addition of the specific MSS planner system (rather than having the patient pick their own), expansion of outcome metrics, and the employment of BrainHQ for cognitive stimulation in our study. The majority of patients approached for the intervention enrolled and completed the study. Those in the coaching classes were largely adherent to the program showing a high rate of class attendance and homework completion.

We hypothesized there may be greater change in the coaching intervention when compared to self-implementation, but we saw no statistically significant differences between interventions in this small study. In a secondary, exploratory analysis evaluating the treatment groups combined, we saw statistically significant improvements with moderate effect sizes in across several relevant outcome measures. The sample as a whole showed improved use of compensatory skills, reduced symptoms of anxiety, and improved informant reported daily functioning sustained for 6 months post-intervention period. There was short-term improvement in physical activity levels, but this was not sustained. It is noted, however, that the post-intervention date occurred in March of 2020, just as the COVID-19 pandemic closed many businesses, including fitness centers and gyms. Thus, the lack of sustained outcome specifically in physical activity minutes may also be related to the pandemic. These findings corroborate those of the study by Denny et al. in that both significantly improved compensatory behaviors (EComp) but had no significant sustained effect on physical activity. Thus, certain brain health behaviors may be easier to implement than others in this population. In addition, the participants had already exercised on average 193 min weekly at baseline, and so further improvement may not have been necessary or feasible for some subjects. While the Denny et al. study demonstrated significantly increased engagement in cognitive activities and meditation, we did not find significant effect sizes for LAQ and MAAS scores. In fact, in our group, there was a trend toward increased cognitive activity in the self-implementation group when reviewing the BrainHQ activity data specifically.

We caution that our sample sizes are very small without an untreated control group for comparison. However, in the spirit of pragmatic clinical trial development, we are hopeful that these preliminary data support the benefit of future studies to better understand the efficacy of these two scalable interventional strategies in larger samples.

The absence of significant differences in change on the primary outcomes between coaching-class and self-implementation is compatible with the idea that motivated patients may not need a formal coaching program to achieve the desired outcome. In fact, in some cases (such as the above mentioned BrainHQ data) there were potential trends toward increased action in the self-implementation group. However, we cannot rule out failure of the coaching intervention as the cause of the lack of significant differences. This trend contrasts with MSS usage in the trial, which was more robust at the end of the treatment period in the coaching group than in the self-implementation group. This was not sustained over the follow-up period, unfortunately. This is consistent with trials by the creators of the MSS, which reported significantly higher MSS usage, functional ability, and memory self-efficacy in the coaching intervention arm compared to the self-implementation control arm of patients with MCI [15]. However, it is noted that use of the MSS has so far focused on the MCI population, where there is objective significant impairment in memory as opposed to the current population of individuals with subjective concerns without objective impairment. Thus, it may be more difficult for those who are objectively cognitively impaired to self-implement than those with subjective impairment. This is consistent with findings from De Wit et al. and Khayoun et al. showing that within the MCI population, degree of cognitive impairment is a primary predictor of successful learning of the MSS [54,55].

Similarly, a multidomain intervention of diet, exercise, and cognitive engagement titled the Body Brain Life in General Practice (BBL-GP) found significantly lower Australian National University-Alzheimer’s Disease Risk Index (ANU-ADRI) scores in the coaching group compared to the self-implementation group of patients with MCI or SCD [56]. However, an internet-based BBL-GP intervention combined with coaching yielded significantly lower Australian National University Alzheimer Disease Risk Index Short Form (ANU-ADRI-SF) scores than the internet-based intervention alone in patients who were overweight or had a chronic medical condition but did not have cognitive impairment [57]. Notably, the BBL-GP intervention did not incorporate a formal bookkeeping or calendar component, which may underlie the benefit of coaching sessions. If our findings can be reproduced in larger scale studies, the operationalization of brain health interventions in the form of self-guided EMS-based programs may be a highly cost-efficient treatment option for certain patients with the earliest signs of cognitive impairment, when implementation may require less resource utilization.

Of the six contacted patients who declined to enroll in the study, four cited concerns over time requirement if they were randomized to the coaching arm. Moreover, one of the participants withdrew from the study due to scheduling conflicts. This indicates that even if larger studies with more power were to detect significant benefits of coaching-class over self-implementation, the self-implemented program may be much more accessible to the target population. Attending 2 h of group sessions per week may be prohibitively inconvenient for some patients, especially those who have work or family commitments. Interestingly, participants in the self-implementation arm spent more time on average on BrainHQ, which provides high levels of patient engagement and instant feedback. Perhaps the combination of the organizational aspect of the EMS system and the interactive nature of BrainHQ forms an effective clinical program for motivated and independent individuals who would prefer to work at their own pace to apply clinician provided brain health recommendations.

The study adds to the growing body of research on multidomain interventions for preventing and delaying cognitive decline. The largest multidomain trial, the FINGER study, found a 2-year diet, exercise, cognitive training, and vascular risk monitoring intervention involving coaching sessions to lead to significantly higher neuropsychological test battery scores in older adults with cognitive performance at or slightly below levels expected for age compared to the control group receiving general health advice [27]. These seminal findings warrant the search for methods to operationalize this knowledge to clinically promote brain health. The recent Brain Health Champion study demonstrated a motivational-interviewing intervention significantly improving physical activity, adherence to a Mediterranean diet, social/cognitive stimulation, and quality of life in patients with SCD or MCI [58]. Although the study lacked a formal calendar or organizational system, it highlighted the importance of goal setting, which was incorporated into the EMS component of our program. Furthermore, a multidomain intervention involving physical exercise, cognitive training, nutritional advice, and health education delivered through periodic phone calls significantly improved cognitive performance in patients with physio-cognitive decline syndrome [59]. Thus, the efficacy of such interventions has also been demonstrated in other pathologies. Our study evinces the utility of the EMS system to protect cognition in patients with SCD. Together with the trial by Denny et al., the results suggest that a multidomain intervention that fundamentally targets the functional deficits associated with cognitive decline may be an effective treatment paradigm for SCD.

Limitations of the study include extremely small sample size, missing cognitive data (HVLT, COWAT, and Symbol Digits) due to lack of in-person appointments during the COVID pandemic, and lack of participant blinding. Considering the number of patients who declined enrollment and withdrew from the study, there may have been selection bias in that active participants tended to be highly motivated to take action to treat their SCD. Thus, external validity of the results is limited by patient attitude and motivation regarding brain health. The limited power of the study may not have been able to reveal differences between the intervention and self-implementation treatments if there were any. Furthermore, there is substantial evidence for the positive effects of diet intervention on brain health [60], which was not included in our program. Nevertheless, the promising results warrant larger future trials with more power and the inclusion of a diet modification component. To more closely investigate the merits of self-implementation, a future trial should allow participants to choose whether to attend the coaching program. In addition to the outcomes of such a trial, the association of demographic factors (such as age, education, occupation) with choice of trial arm would be of great interest for further tailoring of a customizable clinical program.

## 5. Conclusions

This small pilot study demonstrates a potential route to a scalable clinical brain health program that is both accessible and cost-effective. We provide further evidence for the concept of targeting the functional limitations posed by cognitive decline by empowering patients with compensatory tools to improve overall brain health and quality of life. The promising results embolden our advocacy for the establishment of standard of care for SCD in the primary care setting. We encourage other clinicians and researchers to invest in the development of truly scalable ways to help patients with SCD to practically take action to improve function and brain health. We expect that future, larger scale studies can provide higher level evidence of efficacy in this approach, and further clarify if certain patients can successfully self-implement after being provided these tools either in a consultation or on-line modules or an app, with or without additional coaching. In conclusion, we hope that this study leads to greater levels of intervention during the critical window of opportunity during the earliest stages of cognitive decline.

## Figures and Tables

**Table 1:** Demographics of active participants.

	Coaching-Class (N = 7)	Self-Implementation (N = 10)	Total (N = 17)
**Age**			
Median (Q1, Q3)	63.5 (62.1, 75.0)	68.8 (63.2, 73.3)	67.6 (62.6, 74.2)
Range	51.5–88.4	57.0–75.9	51.5–88.4
**Gender**			
Male	2 (28.6%)	4 (40.0%)	6 (35.3%)
Female	5 (71.4%)	6 (60.0%)	11 (64.7%)
**Number of Years of Education**			
Median (Q1, Q3)	16.0 (14.0, 18.0)	16.0 (16.0, 17.0)	16.0 (14.0, 18.0)
Range	14.0–20.0	12.0–20.0	12.0–20.0
**Current Employment Status**			
Employed full-time	4 (57.1%)	2 (20.0%)	6 (35.3%)
Employed part-time	0 (0.0%)	2 (20.0%)	2 (11.8%)
Unemployed due to disability	0 (0.0%)	1 (10.0%)	1 (5.9%)
Retired	3 (42.9%)	5 (50.0%)	8 (47.1%)
**Race/Ethnicity**			
Hispanic or Latino	0 (0.0%)	1 (10.0%)	1 (5.9%)
White/Caucasian	7 (100.0%)	9 (90.0%)	16 (94.1%)
**Marital status**			
Married	5 (71.4%)	8 (80.0%)	13 (76.5%)
Living with someone in a committed relationship	0 (0.0%)	1 (10.0%)	1 (5.9%)
Divorced	2 (28.6%)	0 (0.0%)	2 (11.8%)
Never married	0 (0.0%)	1 (10.0%)	1 (5.9%)

**Table 2:** Self and informant-reported outcomes on the primary measures at all timepoints. All active participants (coaching and self-implementation) combined (N = 17).

	Baseline	Post-Intervention	3-Month Follow-Up	6-Month Follow-Up
Physical Activity (Total Minutes)				
Mean (SD)	193.44 (171.36)	263.50 (234.83)	291.71 (378.76)	132.12 (187.28)
Difference from Baseline (95% CI)	-	73.73 (−2.03, 149.50)	112.75 (−38.00, 263.50)	−37.73 (−137.08, 61.61)
*p*-value	-	0.0556	0.1318	0.4289
Effect Size	-	0.54	0.4	0.21
**MAAS Score**				
Mean (SD)	4.52 (0.82)	4.61 (0.54)	4.55 (0.71)	4.38 (0.72)
Difference from Baseline (95% CI)	-	0.09 (−0.19, 0.37)	0.04 (−0.20, 0.27)	−0.11 (−0.43, 0.21)
*p*-value	-	0.5084	0.7501	0.4667
Effect Size	-	0.16	0.08	0.19
**Self-report EComp**				
Mean (SD)	2.05 (0.55)	2.31 (0.37)	2.21 (0.49)	2.24 (0.45)
Difference from Baseline (95% CI)	-	0.27 (0.07, 0.46)	0.16 (0.00, 0.32)	0.20 (0.00, 0.40)
*p*-value	-	0.0096	0.051	0.0521
Effect Size	-	0.71	0.51	0.51
**LAQ Score**				
Mean (SD)	39.88 (8.42)	39.88 (9.77)	40.00 (6.87)	40.19 (7.56)
Difference from Baseline (95% CI)	-	0.00 (−3.82, 3.82)	0.12 (−2.67, 2.91)	1.25 (−2.18, 4.68)
*p*-value	-	1	0.9299	0.4499
Effect Size	-	0	0.02	0.19
**Informant EComp (N = 11)**				
Mean (SD)	1.90 (0.60)	2.43 (0.44)	2.39 (0.67)	2.39 (0.64)
Difference from Baseline (95% CI)	-	0.47 (0.08, 0.86)	0.43 (−0.05, 0.91)	0.40 (0.02, 0.79)
*p*-value	-	0.0227	0.0709	0.0393
Effect Size	-	0.87	0.65	0.76

Effect sizes (Cohen’s d) to measure change from baseline. Statistically significant results are bolded. Abbreviations: MAAS = Mindful Attention Awareness Scale, LAQ = Lifestyle Activities Questionnaire, EComp = Everyday Compensation Questionnaire.

**Table 3:** Self and informant-reported secondary outcomes at all timepoints. All active participants combined (N = 17).

	Baseline	Post-Intervention	3-Month Follow-Up	6-Month Follow-Up
CES-D Score				
Mean (SD)	8.82 (8.59)	8.76 (6.35)	6.38 (5.41)	7.12 (4.47)
Mean Difference from Baseline (95% CI)	-	−0.06 (−4.03, 3.91)	−2.81 (−6.95, 1.33)	−2.06 (−6.17, 2.05)
*p*-value	-	0.9753	0.168	0.3019
Effect Size	-	0.01	0.36	0.27
**REACH**				
Mean (SD)	15.59 (5.79)	14.35 (4.14)	14.75 (4.06)	14.06 (5.52)
Mean Difference from Baseline (95% CI)	-	−1.24 (−4.12, 1.65)	−1.00 (−3.45, 1.45)	−1.75 (−3.25, −0.25)
*p*-value	-	0.3781	0.3972	0.0252
Effect Size	-	0.22	0.22	0.62
**QOL-AD Score**				
Mean (SD)	39.12 (6.53)	39.71 (5.83)	40.50 (6.83)	39.94 (5.46)
Mean Difference from Baseline (95% CI)	-	0.59 (−1.44, 2.61)	0.94 (−1.33, 3.20)	1.56 (−0.52, 3.65)
*p*-value	-	0.5466	0.3915	0.1313
Effect Size	-	0.15	0.22	0.4
**Ryff Total**				
Mean (SD)	253.12 (23.16)	256.29 (19.82)	256.44 (24.81)	255.81 (22.51)
Median (Q1, Q3)	256.00 (230.00, 267.00)	260.00 (250.00, 268.00)	262.50 (237.75, 272.75)	255.50 (242.00, 271.75)
Mean Difference from Baseline (95% CI)	-	3.18 (−4.47, 10.82)	4.00 (−4.27, 12.27)	4.81 (−4.58, 14.20)
*p*-value	-	0.3914	0.3191	0.2919
Effect Size	-	0.21	0.26	0.27
**Informant ECog (N = 11)**				
Mean (SD)	53.10 (11.73)	53.09 (7.82)	51.82 (11.57)	50.91 (8.51)
Mean Difference from Baseline (95% CI)	-	−0.60 (−5.00, 3.80)	−1.50 (−5.32, 2.32)	−2.90 (−5.46, −0.34)
*p*-value	-	0.7647	0.3974	0.0304
Effect Size	-	0.1	0.28	0.81

Effect sizes (Cohen’s d) to measure change from baseline. Statistically significant results are bolded. Abbreviations: CES-D = Centers for Epidemiological Studies-Depression, REACH = Resources for Enhancing Alzheimer’s Caregiver Health (Anxiety Inventory Form), QOL-AD = Quality of Life-Alzheimer’s Disease, ECog = Everyday Cognition.

**Table 4:** Cognitive outcomes at all timepoints. All active participants combined.

	All Active Subjects Outcomes			
	Baseline	Post-Intervention	3-Month Follow-Up	6-Month Follow-Up
**HVLT Total Recall**				
N	17	7	14	13
Mean (SD)	47.47 (9.15)	48.14 (7.56)	49.50 (9.77)	51.31 (9.30)
Median (Q1, Q3)	49.00 (40.00, 52.00)	50.00 (47.00, 52.50)	47.00 (43.25, 53.25)	51.00 (42.00, 60.00)
Mean Difference from Baseline (95% CI)	-	−0.43 (−9.55, 8.69)	3.71 (−0.63, 8.06)	5.00 (−0.10, 10.10)
*p*-value	-	0.9122	0.0878	0.0539
Effect Size	-	0.04	0.49	0.59
**COWAT Total Items**				
N	17	7	14	13
Mean (SD)	11.94 (2.56)	12.14 (2.41)	11.36 (2.59)	12.31 (2.98)
Median (Q1, Q3)	12.00 (11.00, 14.00)	12.00 (10.50, 12.50)	11.00 (10.00, 13.00)	13.00 (12.00, 13.00)
Mean Difference from Baseline (95% CI)	-	−0.57 (−1.86, 0.72)	−0.29 (−1.26, 0.69)	0.38 (−0.49, 1.26)
*p*-value	-	0.3208	0.5365	0.3563
Effect Size	-	0.41	0.17	0.27
**Symbol Digit Total Items**				
N	17	7	14	13
Mean (SD)	49.76 (8.70)	49.43 (9.78)	54.07 (9.38)	54.54 (10.45)
Median (Q1, Q3)	52.00 (49.00, 53.00)	52.00 (44.50, 53.00)	52.50 (49.25, 59.00)	56.00 (49.00, 58.00)
Mean Difference from Baseline (95% CI)	-	2.14 (−0.85, 5.14)	5.43 (1.12, 9.74)	5.92 (0.96, 10.88)
*p*-value	-	0.1304	0.0175	0.0232
Effect Size	-	0.66	0.73	0.72

Effect sizes (Cohen’s d) to measure change from baseline. Statistically significant results are bolded. The results were limited due to limited in person appointments during the COVID pandemic. Abbreviations: HVLT = Hopkins Verbal Learning Test, COWAT = Controlled Oral Word Association Test.

## Data Availability

Data are contained within the article.

## Supplementary Materials

The following are available online at https://www.mdpi.com/article/10.3390/brainsci11101306/s1, File S1: Recommendations Document Provided to all Participants, Table S1: Intervention vs. Control Change from pre to post in Primary Outcomes, Table S2: Intervention vs. Control Change from pre to 3 months in Primary Outcomes, Table S3: Intervention vs. Control Change from pre to 6 months in Primary Outcomes.
