# Supplementary material for: Compensatory and Lifestyle-Based Brain Health Program for Subjective Cognitive Decline: Self-Implementation versus Coaching
Source: Brain Sci. 2021 Sep 30;11(10):1306. doi: 10.3390/brainsci11101306 (PMC8534077; doi:10.3390/brainsci11101306)
Supplement: Supplementary file 1 [file brainsci-11-01306-s001.zip › brainsci-1332649-supplementary.pdf]

Supplemental Material File S1: Recommendations Packet

Compensatory strategies: Planner system

We recommend that you use an organized planner system to help you achieve the healthy life-style habits we are encouraging in this study. You should have one and only one system. We recommend the Mayo system enclosed here, which is the book with the blue cover. This system was chosen because it is easily portable, it has ample space with the two page per day format, and it has sections for multiple areas of organization including appointments, to-dos, and notes. We recommend paper over technology as this system is easily maintained in the future even as technology changes. Check your planner 2-3 times each day on a regular basis to build the habit. If you build the habit, we will continue to supply you with these planners yearly even after the study is complete.

Planner sections:
Monthly book—two pages per day format

- **Scheduled Events and Appointments:** This section is a place to record upcoming appointments or events that happen at a specific/designated time
- **To Be Done ('To Do' or Task List for the day):** The To Be Done List is often used to record tasks, routines, and things to be done that do not need to happen at a specific time.
- **Notes (Journal):** Here you can record information that you need to remember later on. Similar to a diary, this section can be used for logging important things that happen during the day that are not appointments and not planned tasks.

Yearly section: This monthly view is for the entire year and provides extra organizational pages. The yearly section is for recording future information. At the beginning of each month, you should spend time transferring information you recorded as future plans into the monthly book.

Notations:
✓ When a task is completed, check it off.
→ This is the transfer arrow. If something planned is not completed, move it forward in your planner either to the next day or to a point in the future when you know you can complete it.
☆or * This is the priority star or asterisk. It is used on the left side of the entry to highlight it as something that is especially important for that day. If you have a lot of priority stars on a day, you may need to consider reprioritizing using a numeric list.

Goal: You are encouraged to have entries in your planner each day in each section. Use it daily to help organize the new healthy brain habits we are encouraging.

Compensatory strategies: Task lists

Getting in the habit of creating task lists to plan and accomplish a project will lead to more success in completing small chores and projects as well as long term goals.

Types of 'Task' lists:
Daily or weekly – What are you going to work on this week or today to work towards your long-term goals? What else needs to happen today or this week?
Long term – What you want to accomplish in the next 3-6 months? What are the things that are going to make a huge impact on your life?

Technique

1. First, brainstorm all the steps you need to accomplish in a task--either a small project or a long term project.
2. After brainstorming, break down each goal into smaller tasks one project at a time.
3. Assign yourself to dos based on these steps. Be sure each step is something that can be completed in one day.
4. Spread the steps out realistically but also be sure to consider when you want the task completed and allocate in the schedule appropriately.

Don't try to do everything for a project at once. Be sure to check off the smaller portions as you do them. If you are having trouble getting started, the first step is too big. Consider giving yourself uninterrupted blocks of time for that day's part of the project especially if it requires more focus.

Goals:

1. **Identify at least one goal related to brain health activity and identify smaller steps to accomplish that goal. Complete at least one small step per week until completed.**
2. **Identify at least one long-term goal not related to brain health and identify smaller steps to accomplish that goal. Complete at least one small step per week until completed.**
3. **Use the planner provided to help organize and achieve these steps.**

Compensatory strategies: Functional zones

In addition to using an organized system like a planner, it is also beneficial to create organized functional zones in your home for activities you perform in daily life. Just like using your organized planner on a regular basis and building that habit, you are encouraged to create functional zones and use and maintain them regularly. “A place for everything, and everything in its place.” Examples could include a zone for managing finances, a zone for a hobby, a zone for a volunteer activity, a zone for tools and maintenance supplies, a zone for medication management, and a zone for mail handling.

Technique

1. Identify and make a list of multiple functional zones applicable to your interests and day to day life.
2. Next, list the steps needed to either create a functional zone for that area or to improve an existing functional zone.
3. After a full list is complete, chose one zone to start with and assign yourself at least two steps from that list for the week. It is recommended that you start with the zone that would need the least work to create.
4. Set a goal to complete at least one of those two steps and carry forward a step if it is not completed.
5. When one zone is completed, start another zone with the goal of having at least two steps on this task each week until the functional zones are complete.
6. Aim to have all functional zones completed by March 2020.

Goals:

1. **Identify multiple functional zones applicable to your daily life and make a list of them.**
2. **For each zone, identify at least 3 steps to create or improve that functional zone.**
3. **Gradually transfer these steps to your To Do list. Have at least two steps from these lists planned for each week and aim to complete at least one of those two.**
4. **Use the planner provided to help organize and achieve these steps.**

Activities for cognitive health: Physical exercise

Having a physical exercise routine is beneficial for so many reasons, including cardiovascular health, improved sleep, and emotional health but also for brain and cognitive health. Aerobic exercise (e.g., walking a speed slightly faster than your normal walking speed) is associated with increased brain volume, increase in hippocampal size (an area in the brain important for new memories), and release of brain-derived neurotrophic factor (a protein that helps maintain healthy neurons in the brain).

For brain and cognitive health, we recommend that you build up a regular exercise program of 150 minutes of activity per week. **Please check with your physician to be sure there are no restrictions to the types of exercise programs he or she would recommend or any limitations he or she would suggest in terms of intensity**. If you are not currently active, we would recommend that you start gradually and work your way up to 150 minutes per week by March of 2020.

Here's what a sample plan looks like:
Week 1: Walk (inside or outside) for 5 minutes M/W/F.
Week 2: Walk (inside or outside) for 7 minutes M/W/F.
Week 3: Add 3 minutes to each walk (10 minutes per day M/W/F).
Week 4: Add 5 minutes to each walk (15 minutes per day M/W/F).
Week 5: Add 5 minutes to each walk (20 minutes per day M/W/F).
Week 6: Add one more day of walking, but only for 10 minutes (20 minutes per day M/W/F and 10 minutes on Saturday)
Week 7: Increase your Saturday walk to 20 minutes (20 minutes per day/4 days per week)
Week 8: Add 5 minutes to each walk (25 minutes per day/ 4 days per week)
Week 9: Add one more day of walking (25 minutes per day M/W/F/S and Tuesday)
Week 10. Add 5 minutes to each walk (30 minutes per day/ 5 days per week)

Plan your activity either by scheduling it at a certain time (Walk 9:00-9:30am) or adding it to your To Do list for the day for a certain duration (walk 30 minutes today).

Goals:

1. **Develop a physical exercise program that includes 150 minutes per week of activity. Feel free to choose any activity or activities you like as approved by your doctor.**
2. **Document those activities either by scheduling in advance or noting the activity and duration of the activity after completion.**
3. **Use the planner provided to help organize and achieve these steps.**

Activities for cognitive health: Cognitive exercise

In addition to physical exercise as discussed above, a 2017 report from the National Academy of Sciences concluded that the positive effects of cognitive training or cognitive exercise may delay or slow age related cognitive decline. The definition of cognitive training is very broad and engages cognitive functions such as problem solving, memory and speed of processing. They may be computer-based but can also include new activities such as learning a new language or instrument or hobby. The emphasis is generally on new and challenging experiences one actively engages in (rather than passively absorbing).

Though cognitive exercise can be a variety of activities, we specifically recommend that our patients incorporate BrainHQ into their routine for at least a portion of their activity. BrainHQ is specifically designed to be at the level of challenge YOU need (it adjusts to you) and will get more challenging as you improve. It also utilizes processing speed or time pressure challenge in most, if not all, activities. Thus, this format will automatically give you the important ingredients for cognitive exercise (challenge and speed) without you having to think about it. There have been several studies with the BrainHQ exercises demonstrating cognitive benefit in terms of improvement in the exercises, improvement in related measures of cognitive functioning (such as working memory and processing speed) and improvements in ratings of day to day functioning. To start you off, you have been given a one year subscription to BrainHQ. Please include this as at least a portion of your cognitive activity. You can log on via the instructions below. Your subscription will last the year, and we will be able to see how much you have used the program via a confidential portal.

In addition, feel free to add in other cognitive exercises with an emphasis on new and challenging activities that require you to engage in the process and with an emphasis on activities that have time pressure for at least some portion. Between BrainHQ and other activities, you should aim for 150 minutes per week of activity.

To utilize your BrainHQ subscription, you may utilize iOS (e.g., Ipad) or Android tablets or smartphones or a desktop or laptop computer (or you can move between multiple devices with the same account as you desire). When you use a tablet or smartphone, first download the BrainHQ app on either the Apple or Android system from the App Store or Google Play. Then use your log on and password as provided below. Please do not share your account with others during the duration of the trial. If you wish to continue your subscription after the study is over, you will need to start a new account using your personal email address. The subscription is $14/month or $96/year.

If you complete your BrainHQ training on a computer (laptop or desktop), first go to the website: https://research.brainhq.com

Whatever device you use your log on will be: CognitiveHealthStudy+22@gmail.com
Password: habit2020

Goals:

1. **Develop a cognitive exercise program that includes 150 minutes per week of activity with at least some of that time utilization of your BrainHQ subscription.**
2. **Use the planner provided to help organize and achieve this.**

Activities for cognitive health: Stress Management

Finally, stress is a part of life for all of us. Stress can include positive (e.g., a wedding, buying a home) or negative events (a medical illness, family discord). Either way our bodies react with a flood of cortisol to activate our sympathetic nervous system and our “fight or flight” response. This can be helpful when dealing with an acute stressor (needing to finish last minute preparations for a wedding) because it narrows focus and increases motivation. However, long-term stress and chronic cortisol release is damaging to the body and brain. Therefore, it is important to practice ongoing active coping and resiliency habits. Resiliency can be fostered in many ways such as spending time having fun with friends, developing a gratitude practice (every day may not be a good day, but there is something good in every day) and mindfulness or meditation practice on a regular basis.

Below are a number of resources for relaxation/mindfulness practice that you can try for stress management:

Mayo Clinic AZ Integrative Medicine offers a number of yoga and meditation classes. The meditation classes are free and the yoga and movement classes are typically very low cost. You may sign up for classes on your own here: https://clients.mindbodyonline.com/classic/mainclass. Select Mayo Clinic Integrative Health AZ, continue to site without logging in, and then “classes” tab to see options available.

Mayo Clinic offers *“Relaxation for Your Mind and Body”* online audio exercises

1. Abdominal breathing (4 min).
https://www.mayoclinic.org/patient-education?VID=vid-20147892
2. Passive Muscle Relaxation (31 min).
https://www.mayoclinic.org/patient-education?VID=vid-20147943
3. Evening Sky Guided Imagery (9 min).
https://www.mayoclinic.org/patient-education?VID=vid-20147948
4. Seaside Serenity Guided Imagery (10 min)
https://www.mayoclinic.org/patient-education?VID=vid-20147955
5. Progressive Muscle Relaxation (10 min)
https://www.mayoclinic.org/patient-education?VID=vid-20147957

The link below was made by Mayo Clinic News and includes meditation, chair yoga, gentle yoga and qigong sequences of about 10 minutes each.
https://www.youtube.com/playlist?list=PLSWR1ylG_6JajsJB0XegjWj9H112v9hAX&disable_polymer=true

Mayo Clinic Patient Education Materials:
Mindfulness: Living in the Moment Pamphlet
Living in the Moment DVD (9 min)
Using Relaxation Skills to Relieve your Symptoms Pamphlet

Goals:

1. **Develop a list of active coping strategies. Try some meditation or mindfulness activities from the options above**

2. **Aim for an active coping activity/mindfulness/meditation for 10 minutes 4 days per week by March 2020.**
3. **Use the planner provided to help organize and achieve these steps.**

Supplemental Materials

Table S1. Invervention vs. Control Change from pre to post in Primary Outcomes

	Intervention (N=7)	Control (n=10)	p-value
**EComp**			0.512
Mean (SD)	0.192 (0.391)	0.317 (0.370)	
Range	-0.378 - 0.676	-0.176 - 0.919	
**PA_Total_Minutes**			0.771
N-Miss	0	2	
Mean (SD)	62.143 (120.064)	83.875 (157.576)	
Range	-60.000 - 270.000	-204.000 - 285.000	
**LAQ_score**			0.406
Mean (SD)	-1.857 (4.451)	1.300 (8.957)	
Range	-7.000 - 5.000	-12.000 - 15.000	
**MAAS_score**			0.403
Mean (SD)	0.229 (0.402)	-0.007 (0.636)	
Range	-0.533 - 0.800	-1.200 - 0.933	

Table S2. Invervention vs. Control Change from pre to 3 months in Primary Outcomes

	Intervention (N=6)	Control (n=10)	p-value
**EComp**			0.506
Mean (SD)	0.091 (0.348)	0.205 (0.310))	
Range	-0.351 - 0.568	-0.157 - 0.743	
**PA_Total_Minutes**			0.822
N-Miss	0	1	
Mean (SD)	98.333 (171.017)	134.889 (359.858)	
Range	-30.000 - 360.000	-275.000 - 1005.000	
**LAQ_score**			0.876
Mean (SD)	-0.167 (4.021)	-0.600 (5.835)	
Range	-5.000 - 6.000	-14.000 - 7.000	
**MAAS_score**			0.317
Mean (SD)	0.200 (0.623)	-0.047 (0.339)	
Range	-0.533 - 1.067	-0.733 - 0.267	

Table S3. Invervention vs. Control Change from pre to 6 months in Primary Outcomes

	Intervention (N=7)	Control (n=9)	p-value
**EComp**			0.333
Mean (SD)	0.144 (0.261)	0.320 (0.403)	
Range	-0.297 - 0.432	-0.371 - 0.892	
**PA_Total_Minutes**			0.493
N-Miss	0	1	
Mean (SD)	-2.143 (192.697)	-68.875 (173.663)	
Range	-270.000 - 360.000	-288.000 - 210.000	
**LAQ_score**			0.867
Mean (SD)	1.571 (5.798)	1.000 (7.246)	
Range	-10.000 - 7.000	-8.000 - 13.000	
**MAAS_score**			0.640
Mean (SD)	-0.029 (0.383)	-0.178 (0.748)	
Range	-0.533 - 0.667	-1.733 - 0.600	

Notes: EComp = Everyday Compensation; PA = Physical Activity; LAQ=Lifestyle Activities Questionnaire; MAAS = Mindful Attention Awareness Scale
